# The impact of receptor-binding domain natural mutations on antibody recognition of SARS-CoV-2

**DOI:** 10.1038/s41392-021-00536-0

**Published:** 2021-03-23

**Authors:** Cheng Li, Xiaolong Tian, Xiaodong Jia, Jinkai Wan, Lu Lu, Shibo Jiang, Fei Lan, Yinying Lu, Yanling Wu, Tianlei Ying

**Affiliations:** 1 MOE/NHC/CAMS Key Laboratory of Medical Molecular Virology, School of Basic Medical Sciences, Shanghai Medical College, Fudan University, Shanghai, China; 2 Department of Comprehensive Liver Cancer, The Fifth Medical Center, Chinese PLA General Hospital, Beijing, China; 3 Shanghai Key Laboratory of Medical Epigenetics, International Co-laboratory of Medical Epigenetics and Metabolism, Ministry of Science and Technology, Institutes of Biomedical Sciences, Fudan University, Shanghai, China

**Keywords:** Infectious diseases, Microbiology

**Dear Editor**,

The ongoing COVID-19 pandemic has resulted in over 25.0 million confirmed cases and over 840,000 deaths globally. As the third severe respiratory disease outbreak caused by the coronavirus, COVID-19 has led to much larger infected populations and coverage of geographic areas than SARS and MERS. Such high prevalence of infection has raised significant concerns about the emergence and spread of escape variants, which may evade human immunity and eventually render candidate vaccines and antibody-based therapeutics ineffective. Indeed, some naturally mutated SARS-CoV or MERS-CoV strains from the sequential outbreaks were reported to resist neutralization by the antibodies isolated during the first outbreak^1,2^. Furthermore, a number of natural mutations have already been identified in the spike protein of SARS-CoV-2. Among them, a variant with the D614G mutation has rapidly become the dominant pandemic form probably due to its fitness advantage^3^. Another spike mutation, the N501Y, was first identified in a mouse-adapted strain of SARS-CoV-2^4^, and also occurred recently in natural human infections. Therefore, it is essential to continuously monitor the emergence of SARS-CoV-2 spike mutations and their potential roles in viral escape from existing neutralizing antibodies.

To analyze the SARS-CoV-2 mutations, we retrieved all the 101,131 full-length SARS-CoV-2 nucleotide sequences uploaded in the GISAID database (https://www.gisaid.org) up to September 15, 2020. We focused on the receptor-binding domain (RBD) of SARS-CoV-2 spike protein, due to the fact that RBD is the most dominant antigenic site for inducing SARS-CoV-2 neutralizing antibodies and contains the majority of neutralizing epitopes^5^. After filtering out ambiguous sequences, a total of 94,079 full-length SARS-CoV-2 RBD sequences were obtained and aligned with the Wuhan-Hu-1 strain (GenBank: MN_908947). A total of 216 mutational events have been observed in 169 RBD residues across 5188 sequences, accounting for 87.1% of all amino acids in RBD (169 out of 194 residues). Such mutation rate is comparable to that of SARS-CoV-2 S1 (88.7%, 597 out of 673 residues) and S2 (89.0%, 470 out of 528 residues) subunits (Fig. 1a). Although RBD has undergone intensive mutations, the mutant sequences compromise only a small percentage of the 94,079 available RBD sequences (Fig. 1b, Supplementary Table S1), suggesting that these RBD mutations have not been fixed in viral populations.

**Fig. 1:**
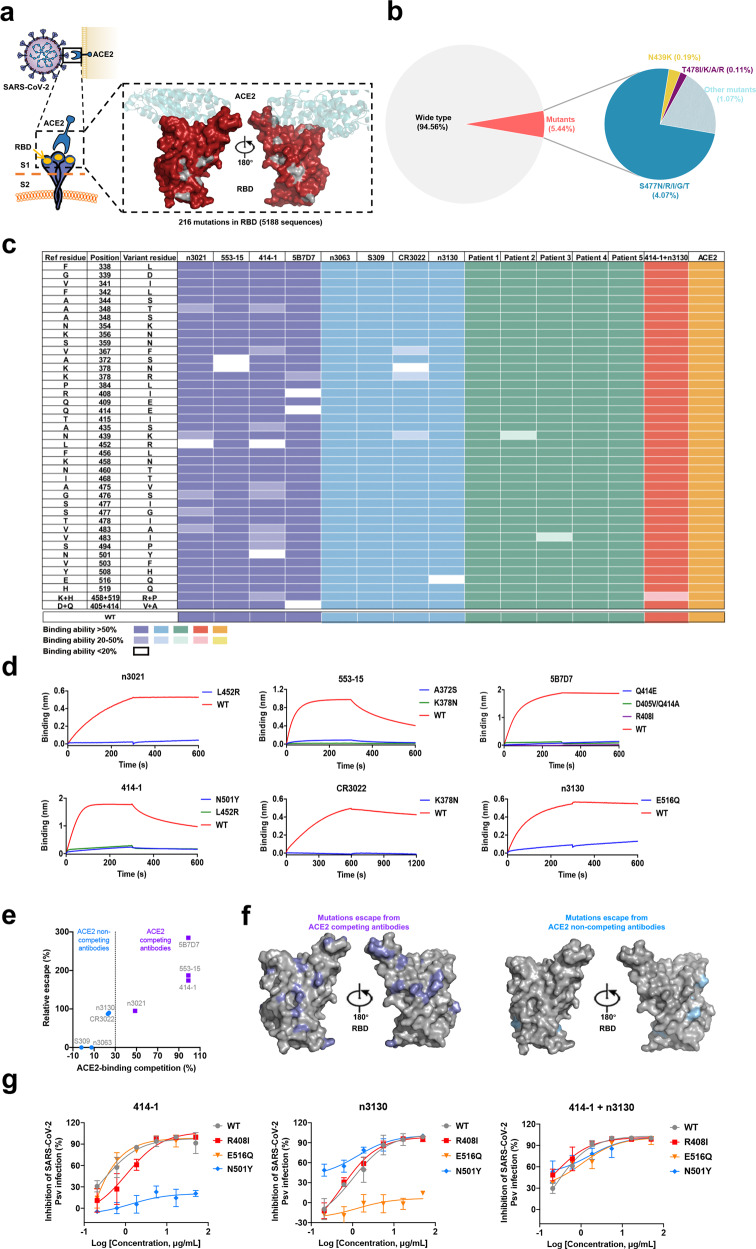
Binding and neutralizing sensitivity of the naturally occurring RBD variants to SARS-CoV-2 binding antibodies and convalescent serum. **a** Surface representations of natural amino acid substitutions in SARS-CoV-2 RBD colored by red. **b** Frequency of the originally reported genome (SARS-CoV-2 Wuhan-Hu-1) and RBD mutants in available RBD sequences at the time of writing (2020). High-frequencies amino acid mutation sites were identified: S477N/R/I/G/T (4.07%), N439K (0.19%), T4781/K/A/R (0.11%). **c** Binding capacity of antibodies against SARS-CoV-2 RBD, human plasma from five COVID-19 convalescent patients, and ACE2 to 41 SARS-CoV-2 RBD mutants, as measured by ELISA. Shades of colors in the boxes indicate retained binding activity of ACE2-competing antibodies (purple), ACE2 non-competing antibodies (blue), convalescent plasma (green), antibody cocktail (red), and ACE2 (orange). Dark color filled boxes indicate the binding ability >50%; light color filled boxes indicate binding ability ranging 50–20%; and white color filled boxes indicate escape to antibodies (binding ability <20%). **d** Escape mutations to antibodies were further confirmed by BLI. **e** Correlation between ACE2 competion values and relative escape of antibodies. ACE2 competion values were obtained from previous reports. **f** Position of binding resistance-conferring substitutions. Structure of the RBD (from PDB 6M17) with positions that are occupied by amino acids whose substitution confers partial or complete (binding ability<50%) escape to antibodies are indicated for ACE2-competing antibodies (purple) and ACE2 non-competing antibodies (blue). **g** Neutralization of luciferase-encoding pseudotyped virus with SARS-CoV-2 S proteins harboring the indicated naturally occurring mutations. Each pseudotyped viruses preincubated with serial dilutions of antibodies or convalescent plasma were used to infect Huh-7 cells, and inhibitory rates (%) of infection were calculated by luciferase activities in cell lysates. Error bars indicate mean±s.d. from three independent experiments

To evaluate the impact of RBD natural mutations on the binding efficacy of anti-SARS-CoV-2 antibodies, we expressed and purified 41 representative RBD variants, which included mutations of the three most frequently-mutated residues (S477, N439, T478), as well as all the variants emerged during the first 4 months of SARS-CoV-2 outbreak. All RBDs expressed well (Supplementary Fig. S1) and retained the capability to bind ACE2 (Supplementary Fig. S2–S4). Then, we measured the binding ability of the RBD mutants to a panel of 8 antibodies, developed by us and other groups that recognize a diverse set of epitopes on SARS-CoV-2 RBD^6–9^. According to the binding epitopes, these antibodies could be divided into two groups: those who recognize epitopes within the ACE2-RBD binding interface and could compete with ACE2 for RBD binding (ACE2-competing group), and those could not (ACE2 non-competing group). Surprisingly, we found that all of the ACE2-competing antibodies exhibited negligible binding to at least one of the mutant RBDs as measured by ELISA (Fig. 1c) and bio-layer interferometry (Fig. 1d). For instance, 414-1, a potent SARS-CoV-2 neutralizing antibody isolated from a COVID-19 recovered patient^7^, exhibited no binding to the RBD mutants L452R and N501Y, and evidently reduced binding to nine other RBD mutants. In contrast, the antibodies S309^8^ and n3063^6^ that engage epitopes distinct from the receptor-binding motif showed exceptional breadth, with no escape mutants observed. The antibodies n3130^6^ and CR3022^9^ were reported to target cryptic epitopes located in the spike trimeric interface, and retained their binding affinities towards most of the RBD variants (Fig. 1c). Taken together, these results indicate that a single natural mutation on the SARS-CoV-2 RBD was able to completely abolish antibody binding. Besides, it seems that the natural RBD mutants had a higher tendency to escape the binding of ACE2-competing antibodies than non-competing antibodies (Fig. 1e and f), although further studies on more extensive panels of antibodies are required to confirm this finding.

Next, we evaluated the binding breadth of a combination of two antibodies recognizing distinct epitopes. Notably, the combination of ACE2-competing antibody 414-1 with non-competing antibody n3130 resulted in full coverage of SARS-CoV-2 RBD variants (Fig. 1c). The mixture exhibited strong binding to most of the RBD variants, and slightly reduced binding only to one double mutant (K448R/H519P). To confirm whether the reduction in RBD binding potency correlates with reduced SARS-CoV-2 neutralization, we also measured the neutralizing activity of the antibodies against SARS-CoV-2 pseudoviruses bearing RBD mutations. As expected, 414-1 and n3130 did not show effective neutralization against pseudoviruses with their corresponding escape mutations (N501Y and E516Q, respectively), while the mixture of the two antibodies broadly neutralized all the tested viruses (Fig. 1g). All the RBD variants pseudoviruses still possessed the infectivity of target cells (Supplementary Fig. S5). In addition, plasma from convalescent COVID-19 patients were also measured for their binding and neutralization activities. Similarly, all plasma samples had superior breadth in binding to naturally mutated RBDs and neutralizing multiple viral variants (Supplementary Figs. S6, S7). Considering that a number of neutralizing antibodies are being developed to treat COVID-19, our results suggest that some of these antibodies should be used in combinations to increase the neutralization breadth and reduce the possibility that an escape mutant is fixed in the treated host population. Nevertheless, the low frequencies of RBD mutants identified here revealed that they are more likely from randomized mutation, and may not represent the result of fitness selection.

Collectively, these results confirmed the capability of SARS-CoV-2 to escape from antibodies, especially the ACE2-competing antibodies, by acquiring resistance mutations in RBD. Such escape mutations can occur within the binding epitopes of antibodies, or regions away from antibody epitopes but may affect immunogenicity of RBD and render antibodies ineffective (Fig. 1f). Notably, the ACE2-competing antibodies constitute the majority of anti-RBD antibodies elicited by SARS-CoV-2 infection or vaccination. Therefore, our findings highlight the importance of continuously monitoring RBD natural mutations and evaluating their impact on antibody recognition of SARS-CoV-2, which may help guide the development and implementation of therapeutic antibodies and vaccines against SARS-CoV-2.

## Supplementary information

Figures. S1 to S7, Table S1
