# Supplementary material for: The impact of receptor-binding domain natural mutations on antibody recognition of SARS-CoV-2
Source: Signal Transduct Target Ther. 2021 Mar 23;6:132. doi: 10.1038/s41392-021-00536-0 (PMC7985591; doi:10.1038/s41392-021-00536-0)
Supplement: Supplementary file 1 — Figures. S1 to S7, Table S1 [file 41392_2021_536_MOESM1_ESM.docx]

**Supplemental Materials**

**The impact of natural mutations in the receptor-binding domain on antibody recognition of SARS-CoV-2**

Cheng Li^a#^, Xiaolong Tian^a#^, Xiaodong Jia^b#^, Jinkai Wan^c^, Lu Lu^a^, Shibo Jiang^a^, Fei Lan^c^, Yinying Lu^b^*, Yanling Wu^a^*, Tianlei Ying^a^*.

*^a^MOE/NHC/CAMS Key Laboratory of Medical Molecular Virology, School of Basic Medical Sciences, Shanghai Medical College, Fudan University, Shanghai, China;*

*^b^Department of Comprehensive Liver Cancer, The Fifth Medical Center, Chinese PLA General Hospital, Beijing, China;*

*^c^Shanghai Key Laboratory of Medical Epigenetics, International Co-laboratory of Medical Epigenetics and Metabolism, Ministry of Science and Technology, Institutes of Biomedical Sciences, Fudan University, Shanghai, China.*

^#^These authors contributed equally to this work.

*Correspondence: Tianlei Ying ([tlying@fudan.edu.cn](mailto:tlying@fudan.edu.cn)), Yanling Wu ([yanlingwu@fudan.edu.cn](mailto:yanlingwu@fudan.edu.cn)), or Yinying Lu ([luyinying1973@163.com](mailto:luyinying1973@163.com))

**Supplementary information include:**

Materials and Methods

Figures. S1 to S7

Table S1

**Materials and Methods**

***Cell lines***

Huh-7 cells and 293 T cells were cultured in Dulbecco’s modified Eagle’s medium (DMEM) supplemented with 10% fetal bovine serum (FBS) in 37°C, 5% CO_2_ atmosphere.

***Human specimen***

This study included five COVID-19 convalescent patients from the Fifth Medical Center, Chinese PLA General Hospital. All COVID-19 patients were confirmed by SARS-CoV-2 nucleic acid test. Blood samples were collected and plasma samples were heat-inactivated in 56ºC for 30 min. All volunteers signed informed consent forms.

***Bioinformatics analysis of mutations***

All the full-length SARS-CoV-2 nucleotide sequences were downloaded from the GISAID database (<https://www.gisaid.org>) and then translated into six open reading frames (ORFs) using SeqKit v0.12.1^1^. These ORFs were merged and filtered by only preserving sequences that contain the conserved RBD sequence “NLVKNK” between residues 532 to 537. Next, the RBD amino acid sequences were extracted with Excel formula of “MID”. PostgreSQL 12.1^2^ was applied to identify RBD mutations and count the frequency of each mutant.

***Three-dimension structural analysis***

Crystal structure of SARS-CoV-2 RBD and ACE2 complex (PDB ID code: 6M0J) were obtained from the Protein Data Bank (PDB). Three-dimension structures of the complex or SARS-CoV-2 RBD alone were then generated by PyMOL 2.4^3^. The naturally mutant residues or escape mutations were highlighted in different colors.

***Site-directed mutagenesis***

The recombinant vector pSecTag2B-RBD encoding SARS-CoV-2 RBD, or pcDNA3.1-S encoding S protein of SARS-CoV-2 from Wuhan-Hu-1 strain (GenBank: MN_908947), were subjected to generate the plasmids with mutations using a QuickMutation™ Site-Directed Mutagenesis Kit (Beyotime Biotechnology, Shanghai, China). Briefly, two complementary primers (15 to 20 nucleotides) containing the desired mutation were designed. Following site-directed mutagenesis PCR, a mutated plasmid containing staggered nicks was generated and the parental vector was digested using *DpnI* restriction endonuclease (NEB, USA). Afterward, the PCR products including nicked vector with the desired mutations were directly transformed into *E. coli* TOP10 competent cells. The recombinant mutant plasmids were verified by DNA sequencing.

***Preparation of RBD mutants***

The recombinant vectors pSecTag2B-RBD and RBD-mutants encoding residue 319 to residue 541 of SARS-CoV-2 and IgG1 Fc tag were transduced into Expi293 cells by PEI method. After 4 days, supernatants were harvested by centrifuging the culture at 2500 rpm for 15 min and filtering the supernatant with a 0.22 µm vacuum filter. The protein was purified using protein A resin (GE Healthcare). Purity was estimated as 95% by SDS-polyacrylamide gel electrophoresis, and protein concentration was measured using the NanoDrop 2000 spectrophotometer (Thermo Fisher). To determine the integrity of these RBD mutants, costar half-area high binding assay plates were coated with purified recombinant ACE2 at 50 ng/well in PBS overnight at 4°C, and blocked with PBS buffer containing 3% milk powder (w/v) at 37°C. Serially diluted RBDs were added and incubated for 1.5 h at 37°C, and bound RBDs were detected with a 1:8000 dilution of horseradish peroxidase (HRP)-conjugated anti-human IgG1 Fc antibody (Thermo Fisher). The enzyme activity was measured with the subsequent addition of substrate ABTS.

***Antibody expression and purification***

The single-domain antibody n3021, n3063, n3130 and mAb CR3022 in scFv format were produced and stored in our laboratory. SARS-CoV-2 neutralizing mouse antibody 5B7D7 was purchased from Genscript Inc. Human neutralizing antibody 414-1 and 553-15 against SARS-CoV-2 were kindly given by Prof. Fei Lan (Fudan University). For cross-neutralizing antibody S309, the codon-optimal gene coding sequences of heavy and light variable domain (PDB: 6WPS) were synthesized by Genewiz and cloned into pTT expression vectors with human IgG1 and human kappa. The heavy and light chain plasmids were co-transfected in Expi293 cells by PEI at a ratio of 1:1 and were purified using Protein A Sepharose and buffer exchanged into PBS. S309 antibody was analyzed via a standard SDS-PAGE gel to check protein integrity.

***Identification of escape mutations to antibodies***

Costar half-area high binding assay plates were coated with RBD and RBD mutants at 4 ºC overnight. Plates were washed 3 times with PBST (PBS with 0.05% Tween 20) and blocked with PBS containing 5% BSA for 1 h at 37 ºC. Serially diluted antibodies, plasma samples, antibody mixture, and recombinant ACE2 at 5 μg/mL in PBS containing 1% BSA were added and incubated for 1.5 h at 37 ºC. After 5 times wash with PBST, a 1:5000 dilution of horseradish peroxidase (HRP)-conjugated anti-Flag antibody (Sigma-Aldrich), goat anti-human IgG (Fab specific) (Sigma-Aldrich), anti-mouse IgG antibody (Sigma-Aldrich), or mouse anti-His antibody (GenScript), was added for 45 min at 37 ºC. The plates were then washed 4 times again and the ABTS substrate were added. The absorbance at 405 nm (OD_405_) was recorded immediately. The signals obtained from the antibody binding to parental RBD were normalized to 100% binding, and the signals obtained from the antibody binding to RBD variants were expressed as the percentage of the binding activity compared to parental RBD. The RBD variant was defined as “complete escape” from antibody if the binding ability was reduced to less than 20%, and the binding with a reduced level of 20-50% was considered “partial escape”.

***Bio-layer interferometry (BLI) binding assays***

To further confirm that the RBD mutants escape from the binding of antibodies, we measured the binding kinetics by BLI on an Octet-RED96 system (ForteBio). The parental RBD and RBD mutants with an avi-tag in C-termini, were biotinylated using the BirA biotin-protein ligase and immobilized onto streptavidin-coated biosensors. The biosensors were incubated with 5 μg/ml antibodies at 37 ºC for 300 s or 600 s and then immersed into wells containing kinetics buffer. For the binding of the wild-type and 41 representative RBD variants to recombinant ACE2, ACE2 with a His-tag in C-termini was immobilized onto Ni-NTA biosensors. The biosensors were incubated with 5 μg/mL RBD mutants at 37 ºC for 360 s and then immersed into wells containing kinetics buffer for another 360 s. To further determine the K_D_ value of RBD escape mutants binding to ACE2, biosensors immobilized with ACE2 were incubated with three-fold serial dilutions of RBD mutant starting at 100 nM at 37 ºC for 360 s and then immersed into wells containing kinetics buffer for another 360 s. All the curves were fitted by a 1:1 binding model using the Data Analysis software 10.0. K_D_ values were determined by averaging binding curves within a dilution series having R^2^ values of greater than 95% confidence level.

***Establishment and validation of pseudovirus***

Pseudotyped viruses incorporated with spike protein from either SARS-CoV-2, R408I, E516Q or N501Y variant were prepared. 293 T cells were co-transfected with vectors pcDNA3.1-S expressing the spike protein and pNL4-3.luc.RE bearing the luciferase reporter-expressing HIV-1 backbone. After 24 hours, the supernatants containing pseudotyped virus were harvested, filtered (0.22 μm pore size), and stored at −80°C in 2-ml aliquots until use. For titration of the SARS-CoV-2 pseudovirus, a HIV-1 p24 ELISA Kit (Abcam) was used to quantitate. The standard curve was linear progression fitted using Prism (GraphPad) and the amounts of SARS-CoV-2 pseudovirus were calculated. Before performing neutralization assay, serial diluted pseudoviruses were added to Huh-7 cells (10^4^ per well in 96-well plates) for 48 h. Then the luciferase activity in cell lysates was recorded.

***Neutralization assay***

For pseudovirus based neutralization assay, the pseudovirus with relative luminescence unit (RLU) at around 30,000 was selected for further measuring antibody neutralization. Briefly, serial dilutions of antibodies S309, 414-1, n3130, mixture of 414-1 and n3130, and convalescent plasma in DMEM supplemented with 10% fetal calf serum were incubated with pseudoviruses at 37°C for 1 h and then the mixtures were added to monolayer Huh-7 cells (10^4^ per well in 96-well plates). Twelve hours after infection, culture medium was refreshed and then incubated for an additional 48 hours. The luciferase activity was calculated for the detection of relative light units using the Bright-Glo™ Luciferase Assay System (Promega).

**
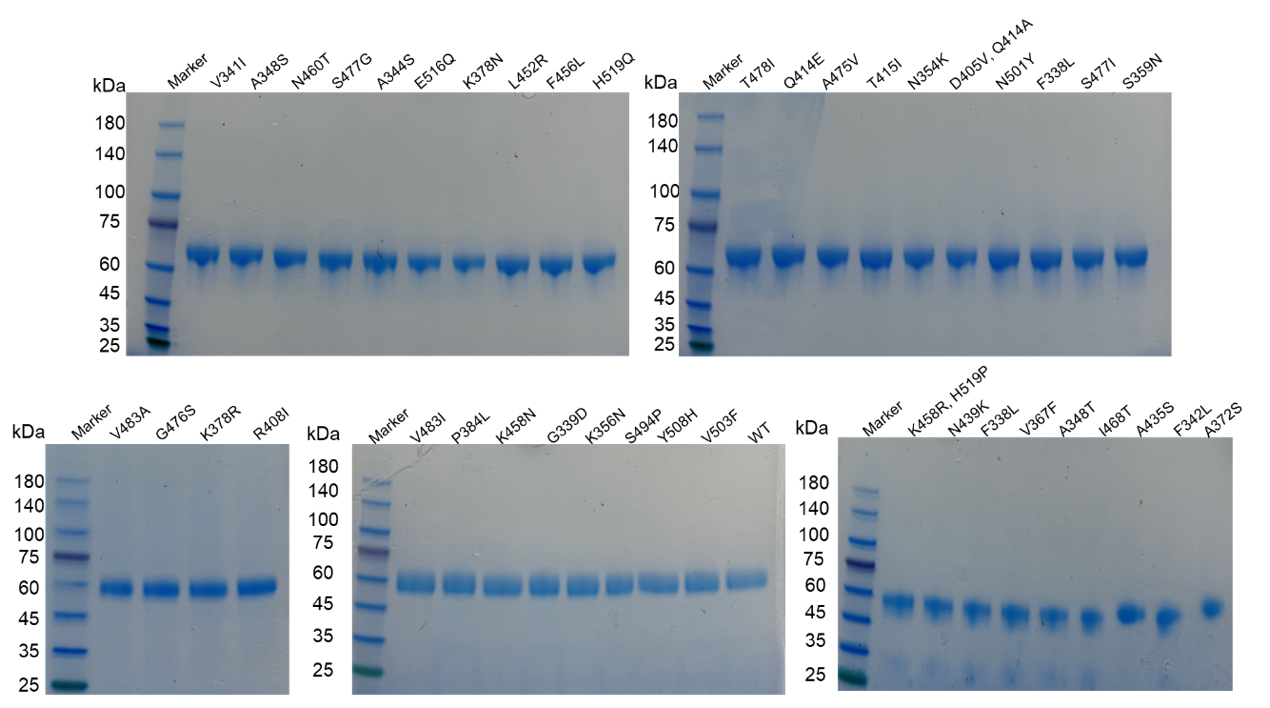
**

**Fig. S1. The purity and integrity of the wild-type and 41 representative RBD variants were determined by SDS-PAGE.**

**
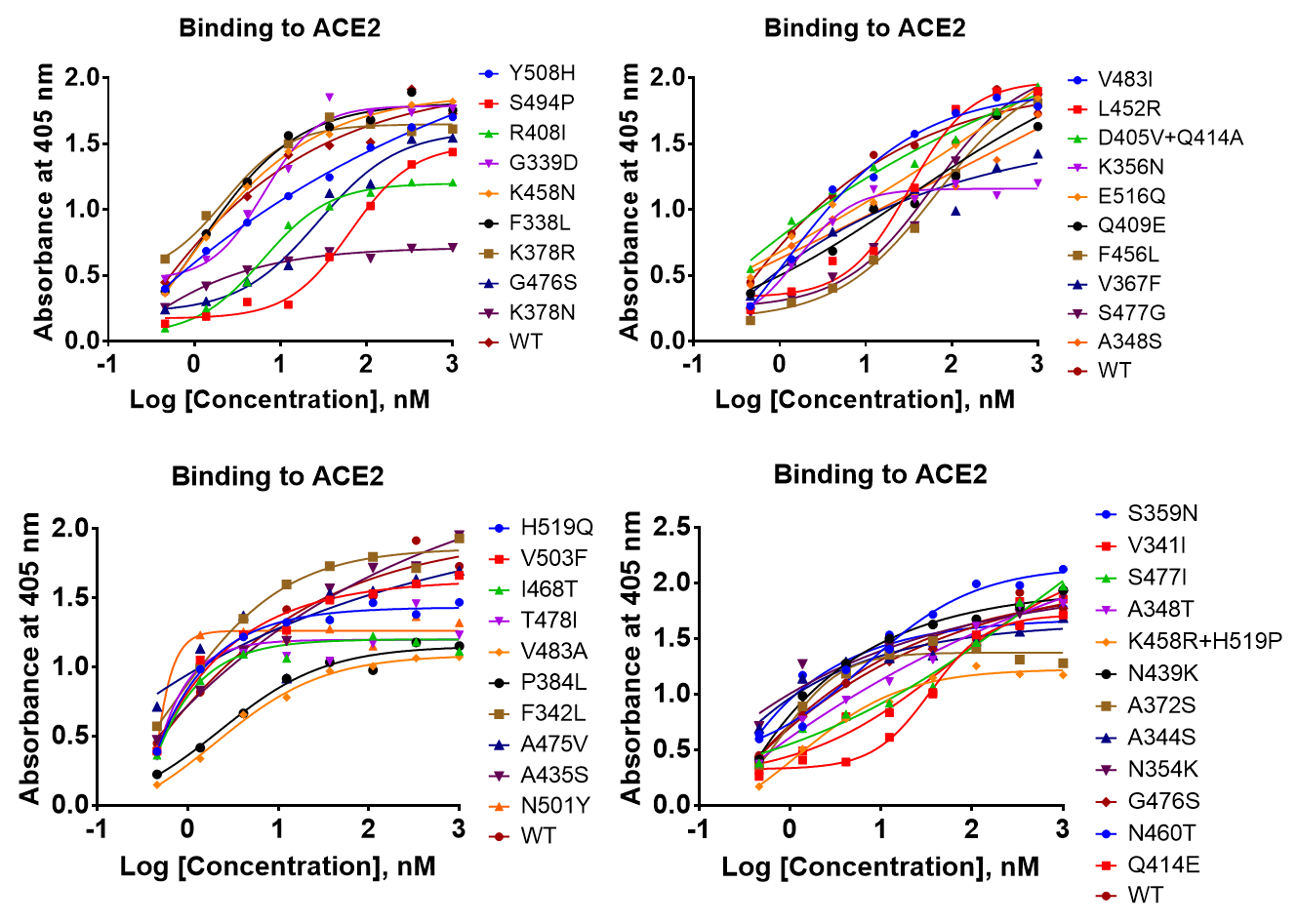
**

**Fig. S2. Binding of the wild-type and 41 representative RBD variants to recombinant ACE2, as measured by ELISA.** Costar half-area high binding assay plates were coated with purified recombinant ACE2 at 50 ng/well in PBS overnight at 4°C, and blocked with PBS buffer containing 3% milk powder (w/v) at 37°C. Serially diluted RBDs were added and incubated for 1.5 h at 37°C, and bound RBDs were detected with a 1:8000 dilution of HRP-conjugated anti-human IgG1 Fc antibody. The enzyme activity was measured with the subsequent addition of substrate ABTS.

**
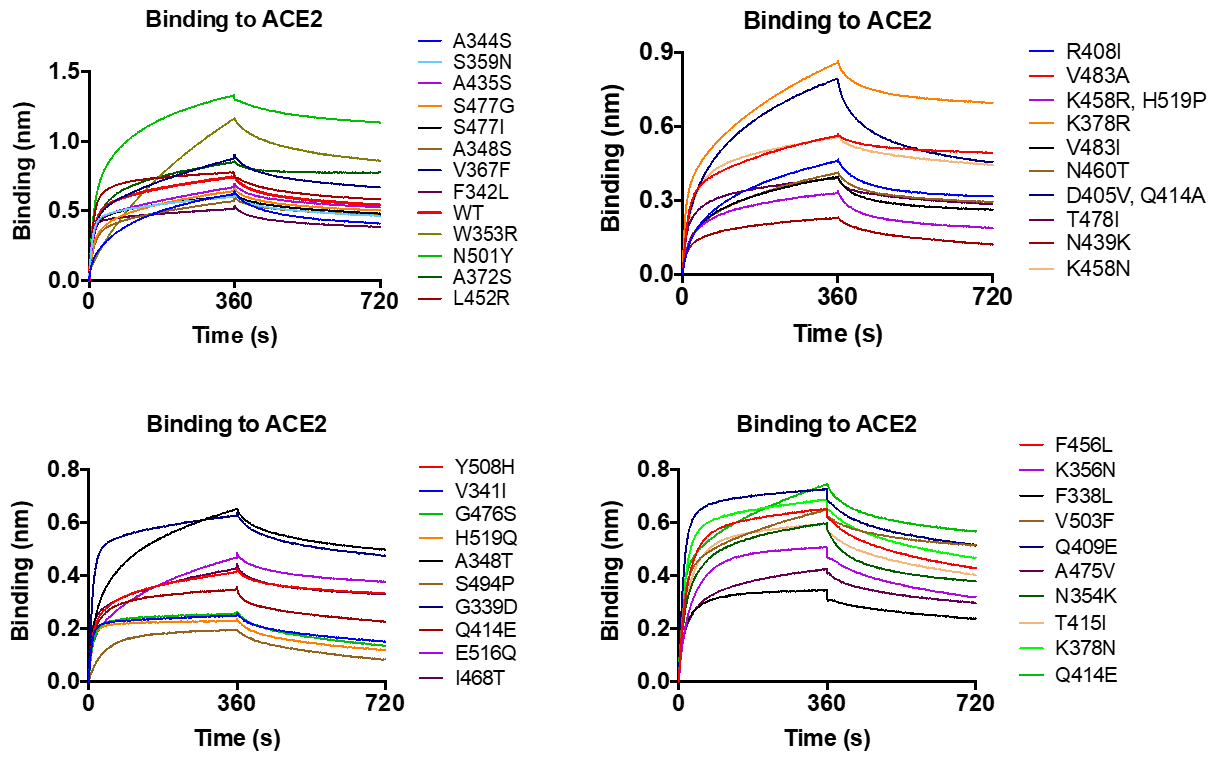
 Fig. S3. Binding kinetics of the wild-type and 41 representative RBD variants to recombinant ACE2, as measured by BLI.** The recombinant ACE2 with a His-tag in C-termini was immobilized onto Ni-NTA biosensors. Then the biosensors were incubated with 5 μg/mL RBD mutants at 37 ºC for 360 s and then immersed into wells containing kinetics buffer for another 360 s. The binding curves were fitted using Prism (GraphPad).

**
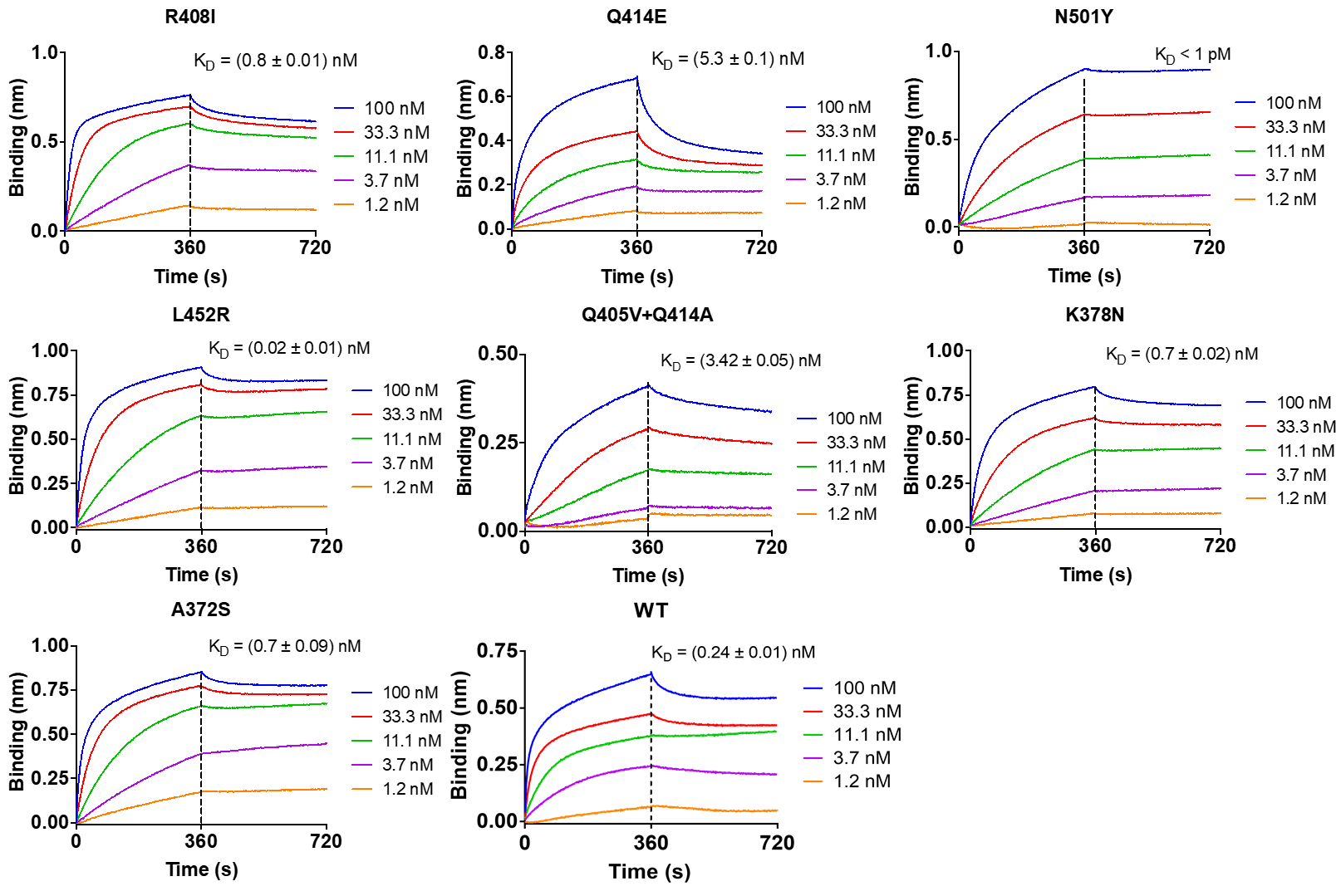
**

**Fig. S4. The binding affinity of the RBD escape mutants to recombinant ACE2, as measured by BLI.** The recombinant ACE2 with a his-tag in C-termini was immobilized onto Ni-NTA biosensors. Then the biosensors were incubated with three-fold diluted RBD mutants starting at 100 nM at 37 ºC for 360 s and then immersed into wells containing kinetics buffer for another 360 s. The K_D_ value was fitted by the Data Analysis software (Forte´Bio).


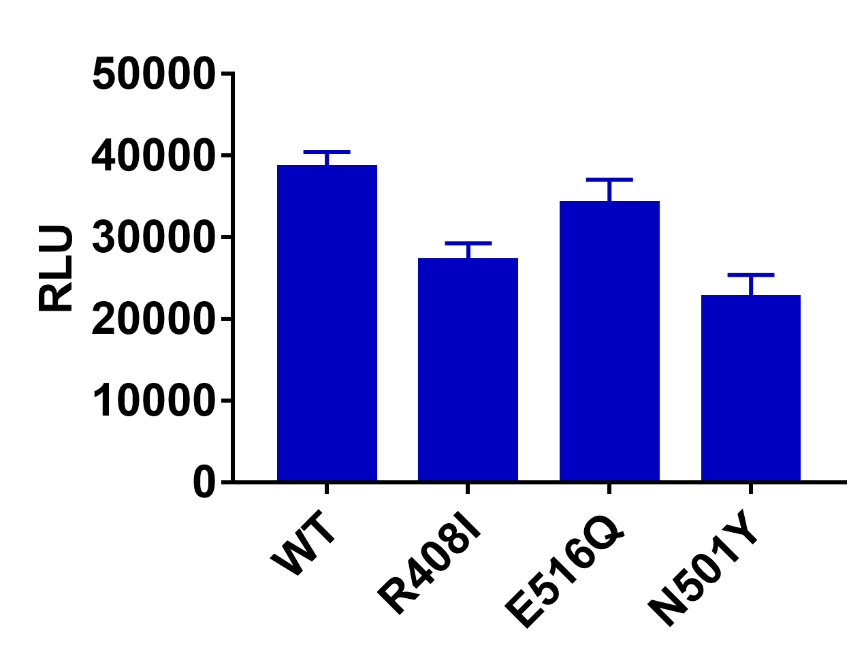


**Fig. S5. Infectivity of SARS-CoV-2 wide-type and RBD variants pseudoviruses.** The amounts of pseudoviruses were measured by HIV1 p24 ELISA Kit (Abcam) following the protocol provided by the manufactures. The standard curve was fitted using Prism (GraphPad) and the amounts were present as pg/ml. The Huh-7 cells were infected by the same titer of pseudoviruses for 48 h and the luciferase readout was recorded. Error bars indicate mean ± s.d. from three independent experiments.


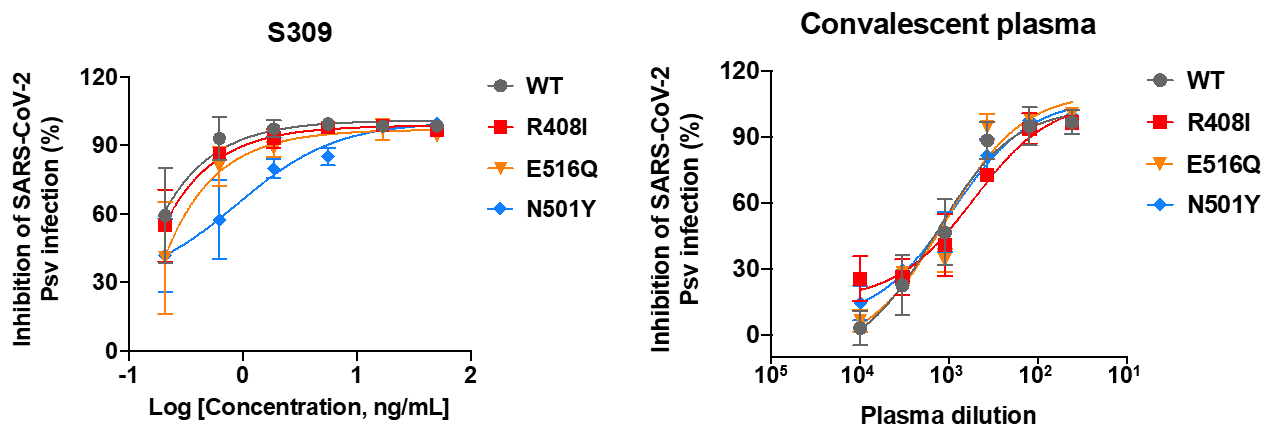


**Fig. S6. Neutralization of luciferase-encoding SARS-CoV-2 pseudotyped viruses harboring wild-type and the indicated naturally occurring RBD mutations.** Viruses preincubated with serial dilutions of antibodies were used to infect Huh-7 cells, and inhibitory rates (%) of infection were calculated by luciferase activities in cell lysates. Error bars indicate mean ± s.d. from three independent experiments.


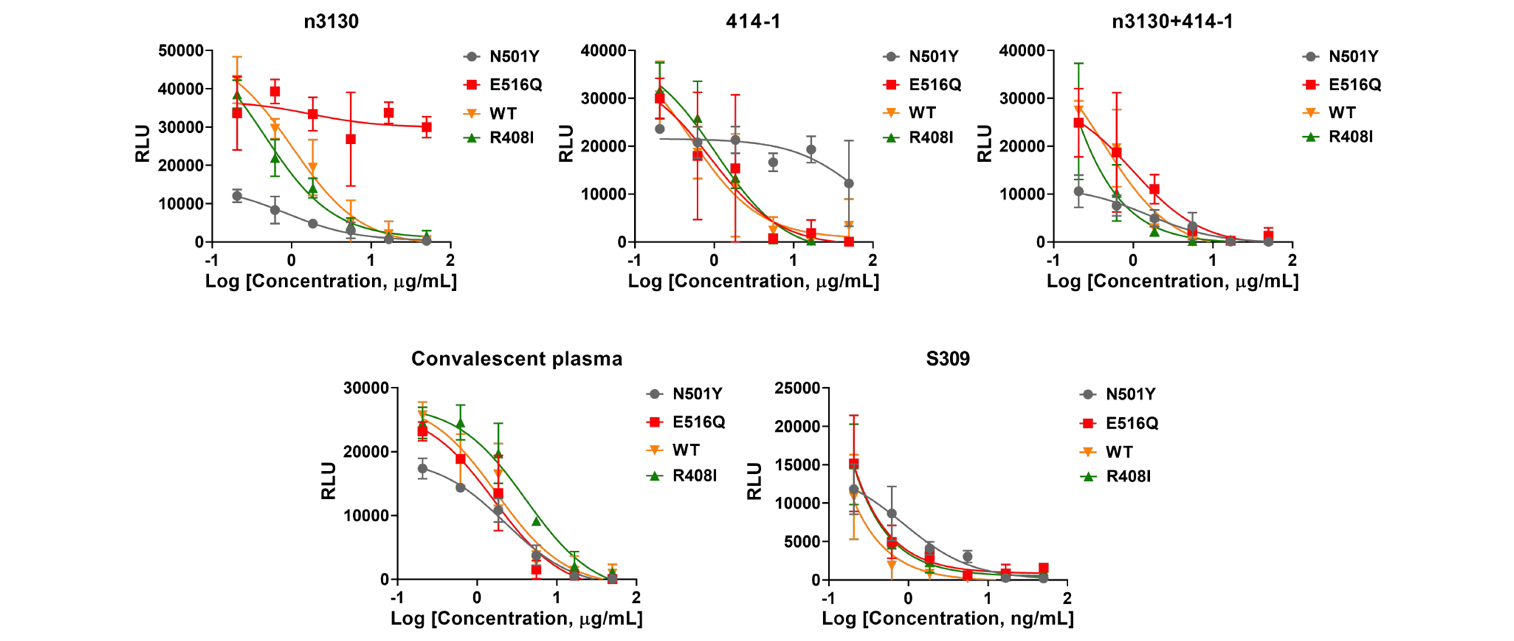


**Fig. S7. The relative luminescence unit of pseudovirus neutralization assay.**

**
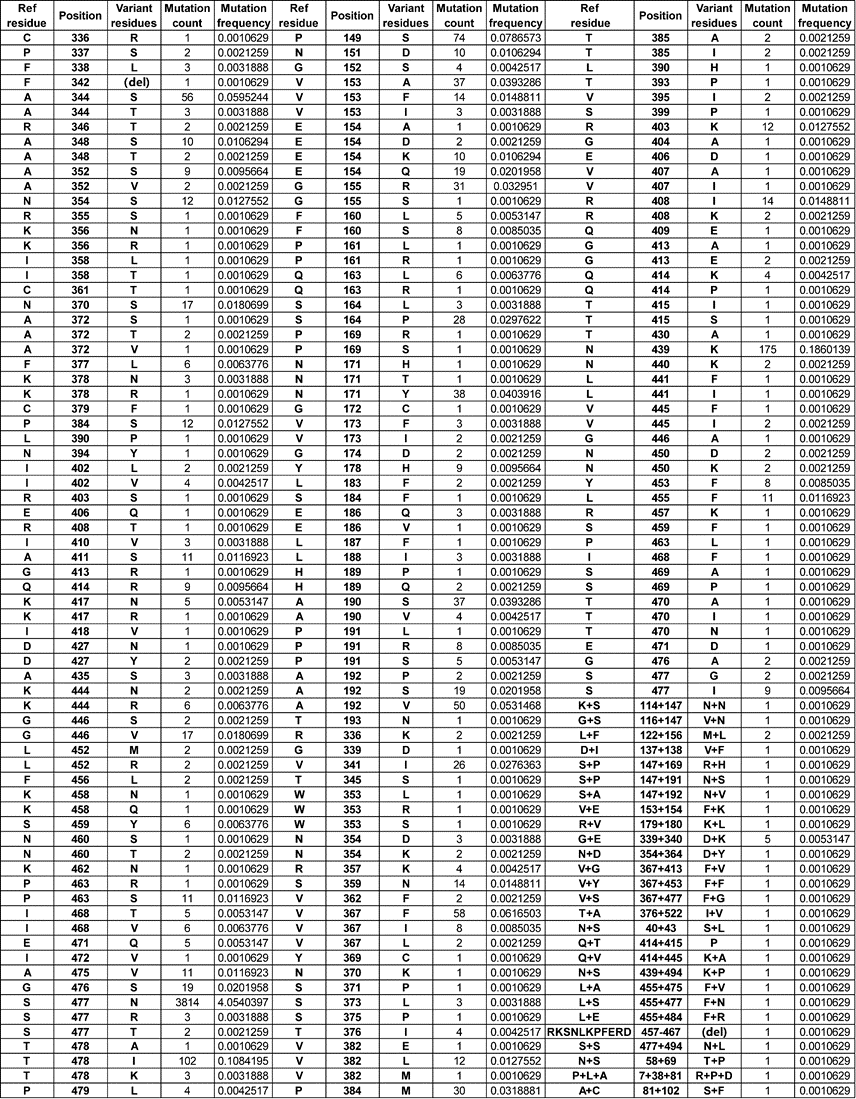
**

**Table S1. All mutations identified with their respective mutation frequencies.**
